# Food Addiction Is Associated with Binge Eating and Psychiatric Distress among Post-Operative Bariatric Surgery Patients and May Improve in Response to Cognitive Behavioural Therapy

**DOI:** 10.3390/nu12102905

**Published:** 2020-09-23

**Authors:** Stephanie Cassin, Samantha Leung, Raed Hawa, Susan Wnuk, Timothy Jackson, Sanjeev Sockalingam

**Affiliations:** 1 Department of Psychology, Ryerson University, Toronto, ON M5B 2K3, Canada; 2 Department of Psychiatry, University of Toronto, Toronto, ON M5T 1R8, Canada; raed.hawa@uhn.ca (R.H.); susan.wnuk@uhn.ca (S.W.); 3 Centre for Mental Health, University Health Network, Toronto, ON M5T 2S8, Canada; samantha.leung@uhn.ca; 4 Bariatric Surgery Program, Toronto Western Hospital, Toronto, ON M5T 2S8, Canada; timothy.jackson@uhn.ca; 5 Division of General Surgery, University Health Network, University of Toronto, Toronto, ON M5T 2S8, Canada; 6 Centre for Addiction and Mental Health, Toronto, ON M5S 2S1, Canada

**Keywords:** bariatric surgery, food addiction, Yale Food Addiction Scale, cognitive behavioural therapy, telephone therapy

## Abstract

The current study examined clinical correlates of food addiction among post-operative bariatric surgery patients, compared the clinical characteristics of patients with versus without food addiction, and examined whether a brief telephone-based cognitive behavioural therapy (Tele-CBT) intervention improves food addiction symptomatology among those with food addiction. Participants (*N* = 100) completed measures of food addiction, binge eating, depression, and anxiety 1 year following bariatric surgery, were randomized to receive either Tele-CBT or standard bariatric post-operative care, and then, repeated the measure of food addiction at 1.25 and 1.5 years following surgery. Thirteen percent of patients exceeded the cut-off for food addiction at 1 year post-surgery, and this subgroup of patients reported greater binge eating characteristics and psychiatric distress compared to patients without food addiction. Among those with food addiction, Tele-CBT was found to improve food addiction symptomatology immediately following the intervention. These preliminary findings suggest that Tele-CBT may be helpful, at least in the short term, in improving food addiction symptomatology among some patients who do not experience remission of food addiction following bariatric surgery; however, these findings require replication in a larger sample.

## 1. Introduction

The increasing prevalence of obesity is a growing global concern [1]. Bariatric surgery remains the most durable intervention for severe obesity, with studies demonstrating significant weight loss and improvements in, or even resolution of, obesity-related comorbidities [2,3,4]. The period of most significant weight loss occurs within the first 12 months following surgery [5,6]; however, between 20% to 50% of patients experience weight regain during long-term follow-up [7,8,9]. Bariatric patients’ weight change trajectories begin to diverge between 6 and 12 months following surgery [5,6] and different trajectories have an impact on the prevalence of comorbidities and corresponding health care costs [10]. Post-operative binge eating, loss of control eating, and grazing have been shown to predict poorer weight loss outcomes following surgery [11,12,13].

The concept of food addiction (FA) may help account for the divergent weight change trajectories observed following bariatric surgery. It has been proposed that certain foods (i.e., hyperpalatable foods with refined carbohydrates and/or added fats) share pharmacokinetic properties with drugs of abuse [14], and are capable of activating an addictive-like process in susceptible individuals that can cause weight-promoting eating behaviours such as compulsive overeating and binge eating [15]. Food addiction is not included as a diagnosis in the Diagnostic and Statistical Manual of Mental Disorders, 5th Edition (DSM-5) [16]; however, the term was first coined in the scientific literature in the 1950s [17] and is widely used by health care professionals, researchers, patients, and the general public. The Yale Food Addiction Scale (YFAS) [18] was developed to operationalize food addiction and identify individuals with addictive tendencies towards highly processed foods. Given the similarities between highly processed foods and drugs of abuse, the YFAS adapted the DSM-IV criteria for substance dependence to make them relevant to the consumption of certain foods such as sweets, salty snacks, fatty foods, and sugary drinks (e.g., consuming more of certain foods than intended or over a longer period of time, preoccupation with certain foods, craving or strong urge to consume certain foods, and continued consumption of certain foods despite knowledge of adverse effects). 

Rates of food addiction in pre-operative bariatric surgery populations as determined by the YFAS [18] and its subsequent modifications range from 14% to 58% [19]. Food addiction has been found to be associated with increased psychosocial impairment, including higher rates of depression, anxiety, impulsivity, and eating psychopathology, particularly binge eating [20,21,22,23,24,25]. In fact, food addiction has been conceptualized as a more severe and compulsive subtype of binge eating disorder (BED) [26]. Although food addiction is positively associated with body mass index [25,27], pre-operative YFAS scores do not appear to be significantly associated with percentage total weight loss (% TWL) following surgery [24,28] and food addiction is not considered a contraindication for bariatric surgery [29]. 

To date, research on food addiction among post-operative bariatric surgery patients remains sparse. Rates of food addiction in post-operative bariatric surgery populations are much lower, ranging from 2% to 14%, with no de novo cases of food addiction identified following bariatric surgery [19]. These improvements in food addiction symptomatology as well as associated problematic eating behaviours have led some researchers to consider whether bariatric surgery could be used as a treatment for food addiction [29]. Despite the improvements in food addiction that generally occur following bariatric surgery, some patients do continue to experience significant food addiction symptomatology. Currently, very little is known about the clinical characteristics of this subgroup of patients; however, they likely represent a subgroup with a particularly severe form of food addiction that may require additional intervention. 

A recent systematic review concluded that there are currently no evidence-based psychosocial interventions for food addiction [30]. To our knowledge, no studies conducted to date have examined therapeutic interventions for bariatric patients experiencing food addiction. However, previous studies have shown that a brief telephone-based cognitive behavioural therapy (Tele-CBT) intervention developed specifically for bariatric surgery patients is effective in improving binge eating, emotional eating, depression, and anxiety among both pre- and post-operative patients [31,32,33]. Given the close association between food addiction, other forms of disordered eating, and psychiatric distress that has been reported in the literature, it is possible that such an intervention may also be effective in improving food addiction symptomatology following surgery.

The current study had three aims: (1) to examine correlates of food addiction among post-operative bariatric surgery patients; (2) to compare the clinical characteristics of patients who meet “diagnosis” for food addiction at 1 year post-surgery to those who do not; and (3) to examine whether Tele-CBT improves food addiction symptomatology among the subset of individuals who meet “diagnosis” for food addiction at 1 year post-surgery. It was hypothesized that: (1) food addiction symptomatology would be strongly correlated with binge eating and moderately correlated with percentage total weight loss and measures of psychiatric distress at 1 year post-surgery; (2) patients meeting “diagnosis” for food addiction would have greater binge eating and psychiatric distress and lower percentage total weight loss compared to those who do not; and (3) patients meeting “diagnosis” for food addiction at 1 year post-surgery who received Tele-CBT would report greater improvements in food addiction symptomatology compared to those receiving standard care.

## 2. Materials and Methods

### 2.1. Study Setting

Patients were recruited between 2018 and 2020 from the University Health Network (Toronto Western Hospital) Bariatric Surgery Program (UHN-BSP) and from the Humber River Hospital Bariatric Surgery Program (HRH-BSP) as part of a larger multisite randomized controlled trial examining the efficacy of telephone-based cognitive behavioural therapy 1 year following bariatric surgery. This study was approved by the institutional Research Ethics Boards and all patients provided informed consent before commencing the study; ethical approval code: CTO #0942. Patients were eligible to participate in the study if they were 1 year post-bariatric surgery, fluent in English, and had access to a telephone and a computer with Internet connection to complete the questionnaires. Study exclusion criteria included active suicidal ideation and poorly controlled psychiatric illness that would preclude engaging in Tele-CBT (e.g., psychosis). Participants were between the ages of 18 and 65 years and had a pre-operative body mass index (BMI) of >40 or ≥35 kg/m^2^ with at least one obesity-related comorbidity. Patients received a Roux-en-Y Gastric Bypass unless a sleeve gastrectomy was surgically indicated (e.g., if there was a history of previous abdominal surgeries resulting in extensive adhesions and/or distorted anatomy). 

### 2.2. Study Procedures

A total of 100 patients completed all study procedures and were included in the analyses. Pre-surgery anthropomorphic data including height and weight were collected by a clinician during pre-surgery appointments. Subsequent post-surgery weights at 1 year post-surgery (henceforth, referred to as “baseline” for the purposes of the present study), post-intervention (1.25 years post-surgery), and follow-up (1.5 years post-surgery) were provided directly from participants via photo or self-report. Percent total weight loss (%TWL) was calculated by dividing the difference in weight at post-intervention by the pre-surgery weight and then multiplying by 100.

All participants completed questionnaires at baseline, post-intervention, and follow-up using Qualtrics (Provo, UT, USA). Upon completion of the baseline questionnaires, participants were randomized to either the Tele-CBT group or the standard care control group using a customized randomization application. Participants randomized to the Tele-CBT group received the intervention described below, whereas those randomized to the control group received standard post-operative care that consisted of routine clinic visits (including post-surgery psychosocial follow-up appointments) and the option of attending a monthly support group. Participants completed questionnaires again at post-intervention and follow-up. The total time interval between the baseline and post-intervention timepoints was 10 weeks.

The Tele-CBT intervention consisted of six 1-hour sessions conducted weekly followed by a 1-hour “booster” session 1 month after the sixth session. Four clinical psychology graduate students who had experience assessing and treating bariatric surgery patients conducted the sessions, and the first author provided clinical supervision. The Tele-CBT intervention introduced participants to a personalized cognitive behavioural model of obesity, and included a variety of clinical strategies such as setting goals, scheduling healthy meals and snacks throughout the day, identifying and planning for difficult eating scenarios, planning pleasurable activities and behavioural alternatives to overeating, engaging in self-care activities, and challenging maladaptive thoughts and solving problems in order to decrease vulnerability to overeating. Participants were asked to complete worksheets for homework between sessions (e.g., food records, thought records) and practice skills that were introduced during the sessions (e.g., engaging in self-care and pleasurable activities) (see full Tele-CBT protocol description) [34]. The final “booster” session served as a check-in for participants to review the skills learned, troubleshoot issues that arose in the month prior, and develop a relapse prevention plan to help maintain the improvements made following surgery.

### 2.3. Study Measures

The Modified Yale Food Addiction Scale Version 2.0 (mYFAS 2.0) [35] was used to assess food addiction symptoms. The mYFAS 2.0 is a 13-item self-report measure designed to assess indicators of addictive-like eating and is comprised of 11 questions assessing substance use disorders as outlined by the DSM-5 and two questions that evaluate clinically significant impairment and distress. Symptom scores on the mYFAS 2.0 range from 0 to 11, and “diagnosis” scores range from no food addiction (1 or fewer symptoms, or does not meet criteria for clinical significance) to mild (2 or 3 symptoms and clinical significance), moderate (4 or 5 symptoms and clinical significance), or severe food addiction (6 or more symptoms and clinical significance). The YFAS 2.0 has been validated in bariatric surgery patients [36], and the abbreviated version of the YFAS 2.0 (i.e., the mYFAS 2.0) has similar psychometric properties as the full version [35]. 

The Binge Eating Scale (BES) was used to assess binge eating symptoms [37,38]. The BES is a self-report measure that assesses the presence of binge eating characteristics suggestive of an eating disorder. It was designed for use with individuals with obesity. Total cores on the BES range from 0 to 46. Moderate and severe levels of binge eating correspond to cut-off scores of 18 and 27, respectively.

The Patient Health Questionnaire-9 (PHQ-9) [39] and the Generalized Anxiety Disorder-7 (GAD-7) [40] were used to assess psychological distress. The PHQ-9 is a self-report measure that assesses depressive symptoms on a scale ranging from 0 (not at all) to 3 (nearly every day). Total scores on the PHQ-9 range from 0 to 27. Mild, moderate, moderately severe, and severe levels of depressive symptoms correspond to cut-off scores of 5, 10, 15, and 20, respectively. The GAD-7 is a self-report measure that assesses anxiety symptoms on a scale ranging from 0 (not at all) to 3 (nearly every day). Total scores on the GAD-7 range from 0 to 21. Mild, moderate, and severe levels of anxiety symptoms correspond to cut-off scores of 5, 10, and 15, respectively. Previous studies have used the PHQ-9 and GAD-7 to assess changes in psychological distress among bariatric surgery patients [41,42,43].

### 2.4. Statistical Analysis

All statistical analyses were performed using SPSS Statistics for Windows (Version 23.0; SPSS, IBM Corp., Armonk, NY, USA). Descriptive statistics including means, standard deviations, and frequency counts were calculated to describe the participant sample. Bivariate correlational analyses were conducted to examine correlates of food addiction. A Pearson’s *r* effect size of 0.1, 0.3, and 0.5 correspond to a small, medium, and large effect, respectively [44]. Change scores were calculated by comparing post-intervention and follow-up to baseline scores. The Shapiro–Wilk test was used to determine whether the data were normally distributed. Kruskal–Wallis H tests were conducted for clinical variables with non-normally distributed data to assesses differences between groups, and Wilcoxon Signed-Rank tests were conducted for clinical variables with non-normally distributed data to assess differences from baseline (pre-intervention) to post-intervention.

## 3. Results

### 3.1. Participant Flow and Characteristics

As mentioned, the current study examining the correlates of food addiction and changes in food addiction in response to cognitive behavioural therapy was part of a larger multisite randomized controlled trial. Of the 136 participants who consented to participate, 122 completed the baseline questionnaires and were randomized to either the Tele-CBT group (*n* = 61) or control group (*n* = 61). Of the remaining participants, eight did not respond to phone calls or emails, two were excluded due to their screening results, and four dropped out due to time constraints. Twelve participants from the Tele-CBT group discontinued treatment due to time constraints and 10 participants from the control group were lost to follow-up due to non-response. Given that this was a pilot study, data analyses for the first and second study aims were conducted with only those who had complete data at baseline (*n* = 100). Participants had a mean age of 48.40 ± 8.51 years, and the majority were female (82%), Caucasian (84%), college or university graduates (68%), were employed full-time (74%), and either married or in a common-law relationship (62%) (see Table 1). Only the subgroup of patients who met “diagnosis” for food addiction at 1 year post-surgery was included in the data analyses for the third study aim (i.e., to examine whether Tele-CBT improves food addiction symptomatology).

### 3.2. Correlates of Food Addiction 1 Year Post-Surgery

The correlates of food addiction are presented in Table 2. As hypothesized, at 1 year post-surgery, mYFAS 2.0 symptom scores were significantly correlated with scores on the BES, PHQ-9, and GAD-7, as well as %TWL. Similarly, mYFAS 2.0 “diagnosis” scores were significantly correlated with scores on the BES, PHQ-9, and GAD-7, as well as %TWL.

### 3.3. Comparison of Participants with versus without Food Addiction 1 Year Post-Surgery

Of the 100 participants in this study, 13 (13%) exceeded the cut-off for food addiction according to the mYFAS “diagnosis” score at 1 year post-surgery. Those who exceeded the cut-off for food addiction reported significantly higher scores on the BES (*p* < 0.001), PHQ-9 (*p* = 0.006), GAD-7 (*p* = 0.027), and mYFAS 2.0 symptoms (*p* < 0.001). Those with food addiction also reported greater %TWL; however, the difference between groups was non-significant (*p* = 0.08). Mean scores (and standard deviations) are presented in Table 3.

### 3.4. Changes in Food Addiction Following Tele-CBT

**Figure 1:**
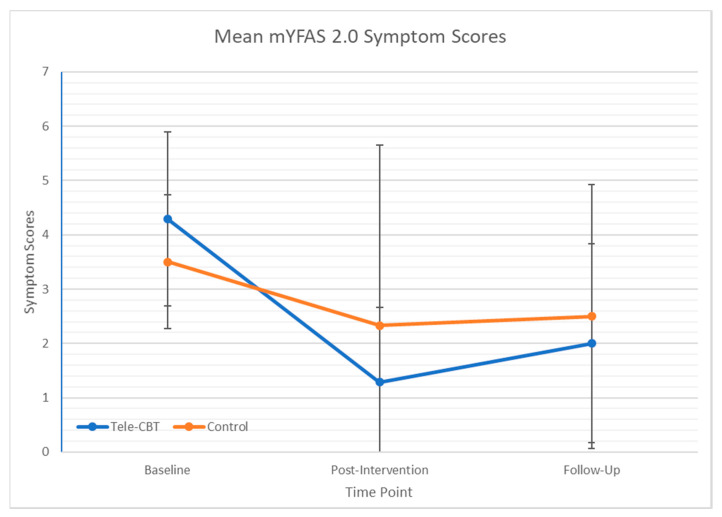
Changes in mYFAS 2.0 symptom scores over time as a function of treatment group among patients meeting “diagnosis” for food addiction at 1 year post-surgery (*n* = 13). Note: Baseline = 1 year post-surgery (prior to CBT); Post-intervention = 15 months post-surgery (immediately following CBT); Follow-up = 18 months post-surgery (3 months following CBT). mYFAS 2.0 Symptomatology—Modified Yale Food Addiction Scale Version 2.0 Symptomatology Scores. Tele-CBT—telephone-based cognitive behavioural therapy.

Changes in mYFAS 2.0 scores were examined among the subgroup of patients who met “diagnosis” for food addiction at 1 year post-surgery (*n* = 13). mYFAS 2.0 symptom scores were significantly lower in the Tele-CBT group (1.29 ± 1.38) than the control group (2.33 ± 3.33) at post-intervention (*p* = 0.027). Patients in the Tele-CBT group reported significant improvements in mYFAS 2.0 symptom scores from pre- to post-intervention (*p* = 0.027), whereas those in the control group did not report significant changes over the same period (*p* = 0.246). mYFAS 2.0 symptom scores were not significantly different between the Tele-CBT group (2.00 ± 1.826) and the control group (2.50 ± 2.429) at follow-up (*p* = 0.772). See Figure 1 for changes in mYFAS 2.0 symptom scores across time as a function of group.

Regarding mYFAS 2.0 “diagnosis” scores, only one patient in the Tele-CBT group met “diagnosis” for food addiction at post-intervention and follow-up. Only two patients in the control group met “diagnosis” for food addiction at post-intervention and they no longer met “diagnosis” at follow-up. However, one other patient reported a resurgence of food addiction despite having remitted at post-intervention.

## 4. Discussion

The current study sought to examine clinical correlates of food addiction among post-operative bariatric surgery patients, to compare the clinical characteristics of patients with food addiction to those without food addiction, and to examine whether Tele-CBT improves food addiction symptomatology among those with food addiction. Our study hypotheses were largely supported. Consistent with the existing literature [20,21,22,23,24,25], food addiction symptomatology was strongly correlated with binge eating characteristics and psychiatric distress (i.e., depression and anxiety symptoms). It was also moderately correlated with %TWL. Thirteen percent of patients exceeded the mYFAS 2.0 cut-off for food addiction at 1 year post-surgery, and this subgroup of patients reported greater binge eating characteristics and psychiatric distress compared to patients without food addiction. They also reported almost 8% less total weight loss on average, though the difference was not statistically significantly likely due to the small sample size and variability in weight loss outcomes. Among those with food addiction, Tele-CBT was found to improve YFAS symptomatology immediately following the intervention. 

The findings of this study add to the literature demonstrating that food addiction tends to improve following bariatric surgery [29]. The rate of mYFAS 2.0 “diagnosis” of 13% in the current study falls within the range of 2% to 14% that has previously been reported among post-operative patients [45,46], and below the range of 14% to 58% reported among pre-operative patients [19]. The mechanisms underlying this improvement are unclear; however, Koball and colleagues [29] identify a number of changes that occur following surgery, including cravings [47], rewarding properties of food [48], food preference and intolerance [49,50], and regulation of hunger and satiety [51,52], which may help account for improvements in food addiction symptomatology. 

The impact of bariatric surgery on food addiction symptomatology is encouraging given the lack of empirical research on treatments for food addiction [30]. The subgroup of patients who meet “diagnosis” for food addiction following bariatric surgery appear to have more persistent and clinically significant binge eating characteristics (falling within the “moderate” range according to BES cut-offs in the current study), which may result in attenuated weight loss outcomes. The very limited research examining the association between post-operative YFAS scores and weight loss outcomes has generated mixed results, with one study reporting that post-operative YFAS score was not associated with the maximum %TWL achieved following surgery but was positively (*r* = 0.22, albeit not significantly, *p* = 0.065) associated with weight regain [53]. 

To our knowledge, this is the first study to examine the effect of a psychosocial intervention on food addiction symptomatology in a bariatric surgery population. Cognitive behavioural therapy has been suggested as a potential treatment for food addiction among bariatric surgery patients, given its efficacy in the treatment of binge eating disorder and substance use disorders [29,30,54]. Patients receiving Tele-CBT reported significant improvements in food addiction symptomatology immediately following the intervention. They improved to a greater extent than those receiving standard post-operative care alone; however, the group difference was no longer significant 3 months following the intervention. These preliminary findings suggest that Tele-CBT may be helpful, at least in the short term, in improving food addiction symptomatology among some patients who do not experience remission of food addiction following bariatric surgery. It is important to highlight that only one patient in each group met “diagnosis” for food addition at the follow-up assessment, which occurred 1.5 years following surgery. Thus, at least among this small group of study participants, it appears that the prevalence of food addiction may continue to decrease between 1 and 1.5 years post-surgery, even among those receiving standard post-operative bariatric care.

## 5. Limitations and Future Research Directions

The results of this study must be considered in light of a number of limitations. First and foremost, a relatively low percentage of patients met “diagnosis” for food addiction at 1 year post-surgery. This is a very encouraging finding; however, it meant that the sample size was very small (*n* = 13) to examine the efficacy of the Tele-CBT intervention among the subgroup of patients with food addiction. It will be important to replicate the findings of this pilot study in a much larger sample of patients with food addiction. If Tele-CBT is found to be effective in improving food addiction symptomatology in a larger replication study, it would be informative to examine the mechanisms of change and predictors of response, similar to studies in patients without food addiction [55]. Second, the Tele-CBT intervention did not have an explicit focus on food addiction. It focused predominantly on goal setting, normalized eating, self-care activities, alternative activities to replace overeating, planning for challenging eating situations, and challenging thoughts that may increase vulnerability for maladaptive behaviours, such as binge eating. It is noteworthy that the Tele-CBT intervention resulted in short-term improvements in food addiction symptomatology despite not explicitly addressing food addiction. However, whether incorporating food addiction into the treatment protocol, using models such as those proposed by Wiss and Brewerton [56] and Treasure and colleagues [57], results in larger and sustained improvements in food addiction symptomatology is an important question that could be empirically tested. Third, given that the intervention was delivered by telephone and thus, did not require patients to travel to the bariatric program, the post-operative weight data used to calculate %TWL were collected directly from patients via photo or self-report, which may impact the reliability of those calculations. Finally, the study sample was quite homogeneous with respect to participant ethnicity (Caucasian) and sex (female). Although this is typical of most bariatric surgery programs, the findings may not generalize to male patients and those of diverse racial and ethnic backgrounds. Regarding future research directions, it would also be informative to conduct a longitudinal study examining pre-operative and post-operative predictors of food addiction symptomatology, as well as the mechanisms that might account for improvements in food addiction symptomatology observed from pre- to post-surgery.

## 6. Conclusions

The findings of this study contribute to the body of literature suggesting that bariatric surgery improves food addiction symptomatology. Patients who continue to experience food addiction after undergoing surgery likely have a more severe form of food addiction that is characterized by greater binge eating characteristics and psychosocial distress. The results of this preliminary study suggest that Tele-CBT may result in short-term improvements in food addiction symptomatology among the subgroup of patients who continue to experience food addiction following surgery.

## Figures and Tables

**Table 1:** Participant Characteristics (*n* = 100).

Variable	M (SD) or *n* (%)
Age (years)	48.40 (8.51)
Gender (female)	82 (82%)
Race/Ethnicity	
Black	4 (4%)
East Asian	1 (1%)
Latin American	3 (3%)
South Asian	1 (1%)
White (Caucasian)	84 (84%)
Other	7 (7%)
Relationship Status	
Married/Common-Law	62 (62%)
Divorced/Separated	13 (13%)
Single	23 (23%)
Widowed	1 (1%)
Occupational status	
Full-Time	74 (74%)
Part-Time	6 (6%)
Retired	7 (7%)
Disability	7 (7%)
Unemployed	6 (6%)
Education	
Some High School	3 (3%)
High School Graduate	7 (7%)
Some College/University	22 (22%)
College or University Graduate	68 (67%)

**Table 2:** Correlates of Modified Yale Food Addiction Scale Version 2.0 (mYFAS 2.0) symptom and diagnosis scores at 1 year post-surgery.

Measure	mYFAS 2.0 Symptom Scores		mYFAS 2.0 Diagnosis Scores	
	r	*p*	r	*p*
BES	0.633	<0.001	0.365	<0.001
PHQ-9	0.459	<0.001	0.217	0.030
GAD-7	0.372	<0.001	0.239	0.016
%TWL	−0.293	0.003	−0.229	0.022

Note: BES—Binge Eating Scale; PHQ-9—Patient Health Questionnaire 9-Item Scale; GAD-7—Generalized Anxiety Disorder 7-Item Scale; %TWL—Percent Total Weight Loss; mYFAS 2.0 Symptomatology—Modified Yale Food Addiction Scale Version 2.0 Symptomatology Scores.

**Table 3:** Clinical characteristics of patients with food addiction (according to the mYFAS2.0), patients without food addiction, and the total sample assessed at 1 year post-surgery. Values represent mean ± standard deviation.

Measure	No Food Addiction (*n* = 87)	Food Addiction (*n* = 13)	Total Sample (*n* = 100)
BES	11.86 ± 7.85	20.46 ± 5.78	12.980 ± 8.12
PHQ-9	5.22 ± 4.60	8.54 ± 3.36	5.65 ± 4.59
GAD-7	4.40 ± 3.96	7.15 ± 4.20	4.76 ± 4.07
%TWL	29.91 ± 9.44	22.09 ± 14.91	28.89 ± 10.55
mYFAS 2.0 Symptomatology	0.72 ± 1.13	3.92 ± 1.44	1.14 ± 1.59

Note: BES—Binge Eating Scale; PHQ-9—Patient Health Questionnaire 9-Item Scale; GAD-7—Generalized Anxiety Disorder 7-Item Scale; %TWL—Percent Total Weight Loss; mYFAS 2.0 Symptomatology—Modified Yale Food Addiction Scale Version 2.0 Symptomatology Scores.
